# Approach to Stereotactic Body Radiotherapy for the Treatment of Advanced Hepatocellular Carcinoma in Patients with Child-Pugh B-7 Cirrhosis

**DOI:** 10.1007/s11864-022-01025-4

**Published:** 2022-11-05

**Authors:** Kayla M. Daniell, Kara Micah Banson, Brett H. Diamond, Shirin Sioshansi

**Affiliations:** 1 Worcester, MA 01605 USA; 2 Hopkinton, MA 01748 USA; 3 Boston, MA 02118 USA; 4 Department of Radiation Oncology, Worcester, MA 01605 USA

**Keywords:** Hepatocellular carcinoma, HCC, Radiation, Stereotactic body radiotherapy, SBRT, Childs-Pugh B7, Liver transplant

## Abstract

Patients with hepatocellular carcinoma (HCC) with underlying Child-Pugh B-7 cirrhosis benefit from management from an experienced, multidisciplinary team. In patients with localized disease who meet criteria for liver transplant, establishing care at a liver transplant center is crucial. For those awaiting transplant, local bridge therapies have emerged as a strategy to maintain priority status and eligibility. Multiple liver-directed therapies exist to provide locoregional tumor control. The careful selection of locoregional therapy is a multidisciplinary endeavor that takes into account patient factors including tumor resectability, underlying liver function, performance status, previous treatment, tumor location/size, and vascular anatomy to determine the optimal management strategy. Technological advances in external beam radiation therapy have allowed stereotactic body radiation therapy (SBRT) to emerge in recent years as a versatile and highly effective bridge therapy consisting of typically between 3 and 5 high dose, highly focused, and non-invasive radiation treatments. When treating cirrhotic patients with HCC, preserving liver function is of utmost importance to prevent clinical decline and decompensation. SBRT has been shown to be both safe and effective in carefully selected patients with Child-Pugh B cirrhosis; however, care must be taken to prevent radiation-induced liver disease. This review summarizes the evolving role of SBRT in the treatment of HCC in patients with Child-Pugh B-7 cirrhosis.

## Introduction

Hepatocellular carcinoma (HCC) comprises the majority of primary liver cancers, which are the sixth most common cancer incidence worldwide and fourth most common cause of cancer-related death [1]. In the USA, the 5-year survival rate for patients with HCC is 18%, and the death rate has increased by 43% from 2000 to 2016 [1]. Therefore, the worsening mortality associated with HCC must be more adequately understood and addressed.

HCC typically develops in the setting of cirrhosis, and the yearly risk of developing HCC ranges from 1 to 5% depending on the cause of cirrhosis [2]. Both HCC and cirrhosis are independently lethal processes, and concomitant cirrhosis is an independent prognostic factor for HCC [3]. Therefore, each should be considered when treating and determining prognosis for patients.

Existing treatments for HCC include hepatectomy, liver transplantation, radio- or microwave ablation, chemoembolization, radioembolization, systemic chemotherapy, and stereotactic body radiotherapy (SBRT) [4]. Though these treatments are intended to relieve tumor burden, they impose inherent toxicity to surrounding liver parenchyma. It is critically important to identify how underlying cirrhosis may impact a patient’s response to HCC treatment.

Tools used to assess liver function include the Child-Pugh (CP) score for Cirrhosis Mortality, the Model for End-stage Liver Disease (MELD) score, and the ALBI (albumin-bilirubin) grade for HCC [4]. The CP score is the most widely used tool in the radiation literature to quantify liver function in patients with HCC. The CP score ranges from 5 to 15 and classifies patients into three classes (A, B, C) based on serum albumin, bilirubin, INR, and subjective measures of ascites and degree of encephalopathy [5]. Scores of 5 and 6 are categorized as CP-A, indicating well-compensated liver function. Scores of 7–9 are categorized as CP-B, indicating borderline liver function [6]. Scores of 10–15 are categorized as CP-C, indicating end stage liver failure. The limitations of the CP classification are the subjective measures of ascites and encephalopathy, and the potential for serum markers to fluctuate due to unrelated physiologic events [7, 8].

Although the CP scoring system classifies patients into three groups, field experts propose that the CP-B class be considered more granularly, as these patients are considered to have borderline liver function and thus their response to treatment for HCC is not well-predicted. Patients with a CP score of B7 (CP-B7) may have compensated cirrhosis, whereas patients with a CP score of B8 or B9 likely have decompensated cirrhosis and may be more adversely impacted by HCC-directed therapies [4].

In this review, we outline the utility of SBRT for the treatment of HCC in patients with CP-B7 cirrhosis by comparing key studies that have included CP-B7 patients. Our hope is to elucidate how treatment of HCC should be approached in patients with “borderline” liver function or those for whom treatment response cannot be well-predicted. Given the lack of consensus and paucity of evidence-based guidelines for determining liver function and planning treatment for patients with HCC, it is imperative that selection of therapeutic modalities be an individualized process, incorporating factors beyond liver function and performance status, especially in the setting of additional comorbidities.

## Treatment

Patients with CP-A liver function have a more favorable prognosis and, consequently, have been the focus of recent reports due to their propensity to tolerate treatment for HCC [4]. Therefore, this group of patients have the most treatment options for HCC available to them. Patients with CP-C liver function have been less well-studied given that they have decompensated liver disease and worse prognosis; therefore, it is presumed that these patients are unlikely to tolerate interventions targeting HCC and thus their care is generally focused on supportive measures [4]. Patients with CP-B liver function, however, have borderline liver function, and while they may not respond to HCC therapies as favorably as patients with CP-A liver function, they will very likely benefit from definitive treatment for HCC. However, predictors of how their liver will respond to HCC therapy are poorly understood and few studies have aimed to identify appropriate therapies for patients with CP-B liver function.

Recent studies of HCC therapies have alluded to the importance of dissecting the CP-B category into its elemental scores: CP-B7, CP-B8, and CP-B9, especially given that this category is bordered by the two extremes of CP-A with a wide variety of treatment options, and CP-C with limited treatments and largely supportive therapies [4, 9–12, 13•]. Specifically, CP-B7 patients may have relatively well-compensated cirrhosis, whereas patients with a CP score of B8 or B9 likely have decompensated cirrhosis with notable ascites, encephalopathy, or jaundice and therefore may be more adversely impacted by treatment for HCC [4]. Therefore, when staging patients and deciding on the most appropriate treatment options for their HCC, it is crucial to consider the factors that contributed to their CP score of B7.

The Child-Pugh model accounts for serum albumin, bilirubin, and INR, as well as subjective measures of ascites and degree of encephalopathy. The minimum possible score is 5, yet a serum albumin measurement of 3.5 mg/dL, despite being within normal limits, adds two points to the CP score and deems the patient a CP-B7. Moreover, an INR > 2.2 in a patient anticoagulated for other medical reasons also deem a patient CP-B7 even with all other factors of the CP model being within normal limits. Thus, it is important to assess the CP score in context of the patient and their confounding or contributory health conditions. Further, treating physicians must recognize that categorization of a patient into CP-B liver function should not be an absolute contraindication to certain therapies for HCC, as they are likely to respond similarly to CP-A patients. Given that most studies of HCC therapies have been studied and approved in CP-A patients, evidence-based guidelines on treatment for HCC in patients with CP-B7 liver function are limited, but we emphasize the importance of a multidisciplinary and individualized approach.

### SBRT for the treatment of HCC

Generally, CP-B classification is an indication for orthotopic liver transplant (OLT), which is the only definitive treatment that will simultaneously cure cirrhosis and HCC. However, only certain patients who are CP-B will benefit from this option, specifically patients who are younger than 67–70, without significant comorbidities, within Milan criteria for liver transplantation, and those who are undergoing downstaging [6]. However, awaiting transplant may take months to years, allowing for the progression of liver disease and HCC to the point where patients are no longer eligible [4]. Therefore, the HCC must be controlled to ensure the patient remains eligible for OLT, and several treatment options exist to mitigate the growth of HCC. One such appropriate treatment for HCC in patients with compromised liver function is stereotactic body radiotherapy (SBRT), in which high-dose radiation is delivered to the tumor over 3–5 treatments. Treatment is planned using a 4D computed tomography (CT) scan taken when the patient is immobilized in a highly reproducible position. The tumor volume is then mapped out based on diagnostic scans and the radiation treatment is planned by a team of radiation oncologists, physicists, and dosimetrists. SBRT is considered to be an appropriate treatment for patients who are awaiting OLT, not eligible for OLT, who have failed previous therapies, or whose lesions deem them ineligible for other therapies [4]. Several studies have shown that SBRT is a safe and feasible option to target HCC in patients with CP-A liver function, and recent studies have shown that select CP-B patients respond to SBRT similarly [14] (see Tables 1 and 2).

**Table 1:** Key trials investigating the safety and efficacy of SBRT in patients with HCC

Study Info	Design	Sample size	CP classification	Radiotherapy dose	Outcomes	Special considerations
Andolino et al. 2011 [11]	Retrospective	*n* = 60 23 underwent OLT	CP-A: 36 (60%) CP-B: 24 (40%) **CP-B7**: **15 (25%)** CP-B8: 6 (10%) CP-B9: 3 (5%)	CP-A: Median dose: 44 Gy in 3 fractions CP-B: Median total dose = 40 Gy in 5 fractions	Median follow-up: 27 months *Response rate: CR: 30% PR: 40% SD: 25% 2-year LC: 90% 2-year PFS: 48% 2-year OS: 67%.	20% experienced progression of CP score within 3 months of treatment. Patients who did not undergo OLT were 2.7 times more likely to experience progression and 17.1 times more likely to die than were patients who did undergo OLT
Culleton et al. 2014 [10]	Prospective	*n* = 29	CP-B: 28 (97%) **CP-B7**: **20 (69%)** CP-C: 1 (3%)	Median dose: 30 Gy in 6 fractions	Median follow-up (median survival): 7.9 months Response rate at 3 months (*n* = 14): PR: 2 SD: 12 Median survival: Entire cohort: 7.9 months CP-B7: 9.9 months CP-B8 and greater: 2.8 months Rate of progression at: 6 months: 30.0% (95% CI: 12.7–47.9) 1 year: 44.5% (95% CI: 22.9–66.1)	Baseline CP score of B7 associated with improved survival (*P* = 0.031) Majority had portal vein thrombosis (76%). Of 16 evaluable patients, 10 (63%) experienced increase in CP index of ≥ 2 points 3 months post SBRT.
Qiu et al. 2017 [15]	Retrospective	(*n* = 93)	CP-A: 50 (53.8%) CP-B: 31 (33.3%) CP-C: 12 (12.9%)	50–60 Gy in 5–10 fractions Dose reductions for patients in which the normal organ dose tolerance was exceeded	Median follow-up: 4.2 months *Response rate (*n* = 82) collected at a median of 87 days after SBRT: CR: 1.2% PR: 35.4% SD: 43.9% PD: 19.5% Median OS: 8.8 months 1-year OS: 38.0% 2-year OS: 29.8% 3-year OS: 21.2% OS by CP class: CP-A: 12.2 months CP-B: 8.4 months CP-C: 4.5 months	Absent or mild treatment-related adverse events: 86% Grade 3 adverse events: 7.5% Grade 4 adverse events: 2.2%
Baumman et al. 2018 [12]	Retrospective	*n* = 37patients with a total of 43 tumors	CP-A5: 15 (41%) CP-A6: 11 (30%) **CP-B7**: **2 (5%)** CP-B8: 4 (10%) CP-B9: 1 (3%) CP-C10: 2 (5%) CP-C11: 2 (5%)	Mean dose = 50 Gy in 5 fractions	Median follow-up: 14 months 42 (97.7%) lesions were completely resolved on MRI by a median of 5.3 months Entire cohort: 12-month LC: 95% No liver progression: 66% OS: 87%	4 patients had grade 3 toxicity All CP ≥ B (mean CP 6.4) had a 1 month break after first three fractions to assess toxicity
Hsieh et al. 2019 [16•]	Retrospective RILD criteria was defined for each patient as well as unirradiated liver volume (ULV) and standard liver volume (SLV). Eastern cohort: Taiwan Western cohort: Texas, USA	*n* = 136	CP-A5: 92 (67.6%) CP-A6: 25 (18.4%) **CP-B7**: **13 (9.6%)** CP-B8: 6 (4.4%)	Median dose (eastern cohort): 66–72.6 Gy in 10–22 fractions Median dose (western cohort): 66–67.5 Gy in 15–20 fractions	Median follow-up: - eastern cohort: 10 months - western cohort: 23 months RILD incidence in CP-A patients (P < 0.001): ULV/SLV ≥ 50%: 0% 49.9–40%: 6% 39.9–30%: 16%, < 30%: 39% RILD incidence in CP-B patients (*P* = 0.006): ULV/SLV ≥ 60%: 0% 59.9–40%: 14% < 40%: 83%	There is a significantly higher risk of RILD in the CP-B group compared with the CP-A group (32% vs. 11%; *P* = .028) ULV/SLV (not mean liver dose) predicted RILD after SBRT.
Jackson et al. 2020 [13•]	Retrospective	*n* = 80	**CP-B7: 37 (46%)** CP-B8: 28 (35%) CP-B9: 15 (19%)	Median dose: 36 Gy in 3 fractions	Median follow-up: 13.1 months Median OS: 17.1 months 1-year OS probability: - CP-B7: 62.2% - CP-B8: 50.0% - CP-B9: 40.0% 2-year OS probability: - CP-B7: 32.4% - CP-B8: 28.6% - CP-B9: 6.7%	Higher treatment dose was associated with better LC, but also larger increases in CP score (18/76 (24%) developed ≥ 2 point CP increase within 6 months of SBRT.
Lee et al. 2020 [9]	Prospective	*n* = 23	**CP-B7: 3 (13%)** CP-B8: 6 (26%) CP-B9: 9 (39%) CP-B10: 5 (22%)	Median dose: 40 Gy in 5 fractions	Median follow-up: 14.5 months (67.1 months in living patients) *Pathologic response rate: CR: 63.6% Median survival: - Entire cohort: 14.5 months - CP-B7 patients: 9.2 months - CP-B8 patients: 22.5 months - CP-B9 patients: 14.5 months - CP-C10 patients: 14.4 months - Patients without subsequent OLT: 7.3 months	10 patients had CP score progression, with 4 patients having a progression of **≥** 2 points by 6 months. No patient had symptoms of RILD.

*HCC* hepatocellular carcinoma, *CP* Child-Pugh score, *SBRT* stereotactic body radiotherapy, *LC* local control, *PFS* progression-free survival, *OS* overall survival, *OLT* orthotopic liver transplant, *GTV* gross tumor volume, *CR* complete response, *PR* partial response, *SD* stable disease, *PD* progressive disease, *PVT* portal venous thrombosis

*Response to treatment per RECIST guidelines (30)

**Table 2:** Key trials investigating SBRT as a bridge to OLT in patients with HCC

Study info	Design	Sample size	CP Classification	Radiotherapy dose	Survival and local control	Drop out rate	Conclusions and special considerations
Sandroussi et al. 2009 [17]	Prospective pilot study	*n* = 10	CP-A5: 4 (40%) **CP-B7: 3 (30%)** CP-B8: 1 (10%) CP-98: 1 (10%) CP-C10: 1 (10%)	Median dose: 33 Gy in 5-6 fractions)	Median follow-up: 8 months *Radiographic response rate: PR: 70% SD: 20% One patient received only 1 fraction before undergoing OLT.	Two patients were de-listed (20%) due to progression of disease outside the treated field and died (lung metastasis; new HCC within untreated segment of liver)	All patients who completed SBRT course had a PR. No grade **≥** 3 acute toxicities CRT did not make transplantation more difficult, nor did it have an adverse effect on the outcome following transplantation.
O’Connor et al. 2012 [18]	Retrospective	*n* = 10 patients with a total of 11 tumors	CP-A: 7 (70%) CP-B: 2 (20%) CP-C: 1 (10%)	Median dose = 51 Gy in 3 fractions	Median follow-up: 62 months *Radiographic response rate at 5 months: PR: 10% SD: 50% 40% of patients underwent OLT before 3 months Pathologic complete response: 3 tumors (27%) 5-year OS: 100% 5-year DFS: 100%	No patients (0%) treated with SBRT dropped off the waitlist due to progression	Four patients experienced acute toxicity, no grade **≥** 3 acute toxicities
Mannina et al. 2016 [19]	Retrospective	*n* = 38 patients with a total of 51 tumors	CP-A: 17 (45%) CP-B7: 21 (55%)	Median dose in CP-A patients: 9-14 Gy in 3-5 fractions Median dose in CP-B7 patients: 8 Gy in 5 fractions	Median follow-up: 4.8 years since OLT *Pathologic response rate: 68% CR: 20 tumors (45%) PR: 10 tumors (23%) SD: 14 tumors (32%) Local recurrence: 9 (24%) Death: 10 (26%) 3-year OS: 77% 3-year DFS: 74%	No patients (0%) treated with SBRT dropped off the waitlist due to progression	PVT in 16% CP score changes from pre-SBRT baseline until pre-OLT: A→A: 34% A→B: 8% A→C: 3% B→A: 8% B→B: 29% B→C: 18%
Moore et al. 2017 [20]	Retrospective	*n* = 23 patients, 16/23 patients treated with SBRT as a bridge to transplant	CP-A: 13 CP-B: 10	Median Dose = 54 Gy	Median follow-up: 12 months *Pathologic response rate: CR: 3 patients (27.3%) PR: 6 patients (54.5%) SD: 2 patients (18.2%) 11 out of the 16 eligible patients underwent successful OLT Median OS and PFS of transplanted patients not reached OS range: 2.0–53.7+ months PFS > 54 months at time of publication	No patients (0%) treated with SBRT dropped off the waitlist due to progression. Remaining 5 patients were awaiting transplant and without disease progression.	Median OS and PFS of non-transplanted patients = 23 months and 14.0 months respectively One patient developed RILD. No other patients experienced decompensation at 12 weeks post-SBRT
Garg et al. 2020 [21•]	Retrospective	*n* = 20 patients	CP-A5: 5 (25%) CP-A6: 5 (25%) **CP-B7: 3 (15%)** CP-B8: 3 (15%) CP-B9: 2 (10%) CP-C10: 2 (10%)	Median dose: 50 Gy in 5 fractions	Median follow-up: 42.4 months in patients who underwent OLT *Radiographic response rate (*n* = 21): CR: 16 (76%) PR: 5 (24%) *Pathologic response rate (*n* = 26): CR: 16 tumors (62%) PR: 10 tumors (38%) Complete clinical response: 76%	Six patients (6/27, 22%) were de-listed: 4 passed away, 2 patients progressed beyond Milan (one developed diffuse carcinomatosis of liver; progression elsewhere in the liver)	No significant correlation between pathologic CR and increasing tumor size (OR = 0.95, 95% CI 0.595–1.53) or equivalent dose in 2 Gy fractions (OR = 1.03, 95% CI 0.984–1.07) Four patients (20%) experienced progression of CP score after SBRT

*HCC* hepatocellular carcinoma, *CP* Child-Pugh score, *SBRT* stereotactic body radiotherapy, *LC* local control, *PFS* progression-free survival, *OS* overall survival, *OLT* orthotopic liver transplant, *GTV* gross tumor volume, *CR* complete response, *PR* partial response, *SD* stable disease, *PD* progressive disease, *PVT* portal venous thrombosis

*Response to treatment per RECIST guidelines [22]

### Safety and efficacy

Stereotactic body radiotherapy has been found to be a feasible, non-invasive approach to treating HCC that is well-tolerated in carefully selected patients with a CP score of A or B [23] (see Table 1). Some studies have begun including select CP-B7 patients in their analyses, and a few of which show that even patients with up to CP-C10 may respond favorably to SBRT [9, 11, 12, 13•, 15]. One feared complication of SBRT is further liver decompensation [10]. Andolino et al. demonstrated that 19.4% of patients with CP-A experienced progression to CP-B, and 20.8% of patients with CP-B experienced progression to CP-C [11]. Furthermore, it reported a relationship between pretreatment CP score and development of any type of toxicity (*P* = 0.035) as well as an increase of more than one grade in hematologic or hepatic dysfunction (*P* = 0.008). Another study showed that SBRT was generally safe in CP-B and C, with a cumulative rate of grade III or higher treatment-related toxicity was 10% versus 3–37% in groups who treated patients of all CP score categories [18]. Although these findings are promising and lay the framework for future research, this data are contrasted by other reports cautioning against irradiating patients with CP-B or C liver function. One study including patients ranging from CP-A5 to CP-B8 reported higher CP classification correlated to a greater risk of developing radiation-induced liver disease (RILD) [10]. Another study reported increased risk of toxicity in patients with CP-B compared to CP-A, and the authors recommended prescription dose reduction and tighter liver dose constraints for CP-B patients undergoing SBRT [11]. Andolino et al. proposed that while CP-A patients undergoing SBRT can safely be treated with 48 Gy in 3 fractions, the prescription dose for CP-B patients should be decreased to 40 Gy in 5 fractions with tighter liver dose constraints [11]. Further, Hsieh et al. recommended that relative and absolute contraindications for untreated liver volume to standard liver volume ratio (ULV:SLV) are < 50% and < 30% for CP-A patients, respectively, and < 60% and < 40% for CP-B patients [16•].

Despite lower prescription doses for patients with CP-B liver function, SBRT has shown to offer comparable local control compared to patients with CP-A, with local control ranging from 65 to 100% [1, 10, 23, 24]. However, this benefit should be considered cautiously in light of the risk for further liver decompensation. Andolino et al. demonstrated that greater GTV, CP-B, and absence of OLT were associated with worse progression-free survival (PFS) (*P* = 0.029, 0.013, and 0.018, respectively) and overall survival (OS) (*P* ≤ 0.001, 0.018, and < 0.001, respectively) [11]. Qiu et al. reported similar conclusions, given that median overall survival after SBRT in their study was 12.2, 8.4, and 4.5 months in patients with CP-A, B, and C, respectively [14]. Therefore, an individualized treatment and careful consideration of risk of progression of liver disease versus HCC allows for the safe and effective treatment of patients with CP-B liver function, especially for CP-B7 patients who have proven to tolerate treatment similarly to patients with CTP-A liver dysfunction [13•].

### SBRT as a bridge to OLT

As mentioned previously, awaiting transplant may take months to years, and both treating HCC and observation pose inherent risks for further liver decompensation. Locoregional therapies including radiofrequency ablation (RFA), percutaneous ethanol injection and transcatheter arterial chemoembolization (TACE) have been the standard of care that “bridge” patients to OLT, not only to help mitigate HCC progression but also preserve liver function [25–28].

Some reports have suggested that SBRT treatment may also be beneficial as an interim bridge therapy (see Table 2). These studies have reported pathologic complete response (pCR) ranging from 25 to 62%, with a de-listing or drop-out rate ranging from 0 to 22%.

One study of patients with CP-B7 to CP-C10 liver function who received SBRT for treatment of HCC reported a median survival of 14.5 months in the entire cohort and 9.2 months in patients with CP-B7 liver function. Among patients in this study who did not subsequently undergo OLT, median survival was 7.3 months. However, median survival in patients who underwent OLT was not yet met at the time of publication, insinuating that treatment with SBRT as a bridge to definitive OLT may significantly improve a patient’s prognosis and life expectancy [9].

Further, Garg et al. demonstrated that SBRT as a bridging modality is not only feasible, but also allows for a clinical complete response (cCR) and pCR comparable to other bridging modalities [21•]. In this study, 20 patients with 26 tumors were treated with SBRT as a bridge to OLT, resulting in a cCR rate of 76% and a pCR rate of 62%. The 1-year, 2-year, and 4-year overall survival (OS) was 94.7%, 84.2%, and 71.7% respectively, with median survival not yet reached at the time of publication. Another study by Moore et al. reported similar findings from their study of CP-A and CP-B patients who were treated with SBRT, some of whom received subsequent OLT [20]. By the time of publication, median OS and PFS of the 11 patients who underwent successful OLT had not yet been reached (OS range, 2.0 = 53.7+ months; PFS: > 54 months). When compared to patients who did not undergo OLT after SBRT, who revealed a median OS and PFS of 23 months and 14 months, respectively, there is clear significance in treating with SBRT for the purpose of controlling HCC whilst maintaining liver function as a patient awaits OLT.

Few studies have directly compared the outcomes between locoregional bridging therapies. Sapisochin et al. reported pCR rates for RFA, TACE and SBRT as 49.2%, 24.3%, and 13.3% respectively with no significant difference in dropout rates [29]. Notably, in this cohort, there was a significantly greater proportion of patients in the SBRT cohort that had poorer underlying liver function (higher MELD scores), larger and greater number of tumors and were outside Milan criteria. Mohamed et al. reported pCR rates for RFA, TACE, SBRT, and y-90 as 60%, 41%, 28.5%, and 94% and with the lowest acute toxicity observed in the SBRT and y-90 cohorts [30].

## Ongoing research studies

Although a large, randomized trial comparing SBRT to other liver directed modalities would be ideal, there are several ongoing studies investigating SBRT in combination with other therapies.

Firstly, there are two key studies investigating the role of immunotherapy in conjunction with SBRT for the treatment of HCC. Investigators at the University of Hong Kong are exploring the role of SBRT in combination with durvalumab for inoperable/unresectable HCC in a phase II single-arm study [31]. Another study from Massachusetts General Hospital is exploring the safety and tolerability of neoadjuvant SBRT with atezolizumab and bevacizumab for treating resectable HCC [32].

Other studies are investigating the role of other procedural interventions in combination with SBRT. The University of Michigan Rogel Cancer Center is exploring the safety of combining Yttrium-90 (Y-90) selective internal radiation therapy (SIRT) with subsequent SBRT, both of which are standard individual modalities for treating HCC [33]. In addition, a similar ongoing study at the Icahn School of Medicine at Mount Sinai is evaluating the efficacy of sequential TACE and SBRT for the control of HCC tumors [34].

Finally, there are studies abroad investigating the role of neoadjuvant SBRT. Researchers at University Hospital in France hypothesize that pre-treatment SBRT prior to surgical resection of HCC may improve outcomes by mitigating seeding in the peritumoral environment [35]. Further, the Shandong Cancer Hospital and Institute is investigating the utility of neoadjuvant SBRT plus programmed cell death-1 (PD-1) prior to hepatic resection in patients with resectable HCC [36].

In addition to efforts made toward understanding the extent to which CP-B patients will tolerate and respond well to certain treatments, these studies investigating combined-modality therapy have the potential to illuminate the breadth of treatment options for these patients.

## Conclusions and Recommendations

Given the paucity of evidence that guides treatment recommendations for patients with CP-B liver function diagnosed with HCC, it is important to expand the current pool of data and further delineate class CP-B liver function into its constituents. Namely, for patients with CP-B7 cirrhosis, it is important for the clinician to contextualize the patient’s CP-B7 liver function by considering the patient’s history as well as what specific factors deem their categorization into the CP-B7 class; such considerations include the time-course to which they progressed to have CP-B7 liver function, whether the patient has had previous HCC-directed interventions, how other liver-directed treatments were tolerated, and other comorbidities or physiologic processes contributing to fluctuating serum markers.

We also highlight that the inclusion of transient serum markers in the CP-B score algorithm may result in inconsistent CP classification. Thus, it may be appropriate to trend the patient’s CP score leading up to diagnosis and over the course of treatment. Further, although the CP score is the most widely used tool to classify liver function in patients with HCC, clinicians should also assess the patient’s MELD and ALBI scores to supplement the CP score. No one tool is perfect for understanding the implications of a patient’s liver function on their likelihood to tolerate treatment, but perhaps in conjunction they may offer a clearer picture of the patient’s liver health.

Until more evidence-based guidance emerges, physicians should utilize a parenchymal-sparing approach when contemplating HCC treatment options. With this in mind, SBRT is not always a patient’s best option, as the efficacy and safety of SBRT depends on tumor anatomy, size, and location; namely, central or peri-arterial tumors may be better candidates for radio-ablation or TACE, while more peripheral tumors in a region that allows for sparing of liver parenchyma and other organs at risk may be better-suited for SBRT. Beyond deciphering the most appropriate options for a patient, it is critically important to use a multidisciplinary approach comprised of colleagues from radiology, interventional radiology, radiation oncology, medical oncology, hepatology, transplant surgeons, palliative care, and social workers. Lastly, individualized, patient-centered care should be central to all discussions pertaining to treatment.
