# Correction to: Approach to Stereotactic Body Radiotherapy for the Treatment of Advanced Hepatocellular Carcinoma in Patients with Child-Pugh B-7 Cirrhosis

**DOI:** 10.1007/s11864-022-01037-0

**Published:** 2022-11-25

**Authors:** Kayla M. Daniell, Kara Micah Banson, Brett H. Diamond, Shirin Sioshansi

**Affiliations:** 1 Worcester, MA 01605 USA; 2 Hopkinton, MA 01748 USA; 3 Boston, MA 02118 USA; 4 Department of Radiation Oncology, Worcester, MA 01605 USA

**Correction to: Current Treatment Options in Oncology**

10.1007/s11864-022-01025-4

The original version of this article, unfortunately, contained mistakes. The 2nd author name in the author group was incorrectly written as Kara Michah Banson. The correct spelling should be Kara Micah Banson.

The original article has been corrected.

